# Leadership and work community – views of graduating dental students

**DOI:** 10.1108/LHS-10-2022-0102

**Published:** 2023-03-28

**Authors:** Tiina A. Tuononen, Milka Kauhanen, Anna Liisa Suominen, Marja-Leena Hyvärinen

**Affiliations:** Institute of Dentistry, Faculty of Health Sciences, University of Eastern Finland, Kuopio, Finland; Faculty of Medicine, University of Turku, Turku, Finland; Institute of Dentistry, School of Medicine, University of Eastern Finland, Kuopio, Finland and Oral Health Teaching Clinic, Kuopio University Hospital, Kuopio, Finland; Language Center, University of Eastern Finland – Kuopio Campus, Kuopio, Finland

**Keywords:** Dental education, Dental student, Interpersonal communication competence, Leadership, Work community

## Abstract

**Purpose:**

This study aims to explore what kind of perceptions dental students at graduation stage have on leadership and work communities, and themselves as leaders and work community members after completing a leadership course tailored for them.

**Design/methodology/approach:**

The research material comprised reflective essays written by fifth-year dental students who had participated in a leadership course. The essays were analyzed using qualitative content analysis.

**Findings:**

Most students reported that they had not considered seeking a leadership position before the course, but their views of leadership had grown more positive as a result of completing the course. Students perceived interpersonal communication competence as the most important factor for leaders, the whole work community and for themselves. They assessed that their biggest strengths were found in this area. The biggest challenges in adapting to a work community concerned the students’ professional identities, which were still taking shape at the time of graduation.

**Originality/value:**

The need for leaders in health-care professions is growing due to ongoing reforms, multidisciplinary teamwork, the development of new technologies and patient demands. Therefore, undergraduate leadership education is needed to ensure that students have knowledge of leadership. Graduating dental students’ perceptions concerning leadership and work communities have not been widely explored. Students’ perceptions of leadership were positive after the course and helped students to realize their own potential in this area.

## Introduction

The need for leaders in health-care professions is growing (Morison and McMullan, 2013; Walsh *et al.*, 2015) due to ongoing social and healthcare reforms, multidisciplinary teamwork, the development of new technologies and patient demands (Brocklehurst *et al.*, 2013; Pihlainen *et al.*, 2016; Fontana *et al.*, 2017; Widström *et al.*, 2019; Grocock, 2020). Leadership is an integral part of dentists’ everyday work alongside with clinical work, as dentists lead patients’ courses of treatment and are in charge of oral health-care teams. In addition, dentists are oral health-care work community members led by their superiors. Leadership can be a potential career choice for a dentist alongside with clinical or research career choices (Tuononen, 2018). Therefore, it is important that students are provided with training to improve their leadership skills already during their university studies (Vikneshan *et al.*, 2019). As concluded by Wali (2018), education on leadership skills introduced in the undergraduate curriculum provides the best opportunity for training students to become future leaders. The teaching of leadership skills can influence the student’s education process and has a potential for positively impacting their performance in the labor market (Mota *et al.*, 2018; D’Assunção *et al.*, 2022). However, leadership in the field of dentistry as well as the views and expectations that dental students have concerning leadership and work communities have received little attention in dental research so far (Walsh *et al.*, 2015; Wali, 2018; Hayes and Ingram, 2019).

Accordingly, the need for undergraduate leadership programs has been widely recognized (Morison and McMullan, 2013; Kiesewetter *et al.*, 2013; Field *et al.*, 2017a, 2017b), and students have been shown to have an interest in and genuine need for leadership training (Victoroff *et al.*, 2008,^2009^; Kalenderian *et al.*, 2013). For example, Vikneshan *et al.* (2019) found out that the majority of students agreed that it is important for dentists to have leadership skills, but most of the students had not participated in any leadership program. While some universities already offer such courses, some of these are only available for a limited number of participants. This may result in selection bias (Kalenderian *et al.*, 2013), as those interested in participating in the training may already tend to take on leadership roles or have a positive view of leadership in general. So far, dental schools’ programs in leadership training seem to have been mainly optional, extracurricular or voluntary leadership development courses based largely on a generic business school model of leadership education (Victoroff *et al.*, 2009; Kalenderian *et al.*, 2013; Wardman *et al.*, 2017; D’Assunção *et al.*, 2021). However, it is important to be aware that both newly graduated dentists and their more experienced colleagues have considered the level of knowledge and leadership skills of graduates as limited (Koivumäki *et al.*, 2017; D’Assunção *et al.*, 2022). This imbalance in the supply and demand of competent leaders could be resolved by providing sufficient training in all stages of education and professional life (Morison and McMullan, 2013; Alestalo, 2015; Walsh *et al.*, 2015). Therefore, it would be most beneficial to have universal student participation in a course addressing leadership tailored for dentists (Wardman *et al.*, 2017; Wali, 2018).

Even though there is a lack of studies evaluating the effectiveness of leadership training courses in dentistry (Kumar *et al.*, 2020), previous studies have shown that providing education on leadership promotes students’ interest and curiosity in becoming effective leaders (Kalenderian *et al.*, 2010; Taichman *et al.*, 2014). D’Assunção *et al.* (2021) concluded that a leadership course organised close to graduation could be relevant to the students as they tend to be more open to also learning about nonclinical topics at this point. There is wide agreement that, for many students, leadership development is best accomplished through a set of practical learning methods (Skoulas and Kalenderian, 2012; Kalenderian *et al.*, 2013; Wardman *et al.*, 2017; Ayn *et al.*, 2020). For example, case studies and discussions have supported students’ development for ethics-based problem-solving, and they have gained a better understanding of their leadership skills via role-play, self-reflection and simulations (Skoulas and Kalenderian, 2012; Wali, 2018; McCunney *et al.*, 2019). The social relationships and interpersonal communication development are important core values shared by these programs, and each places an emphasis on individuals working together in inter- and intraprofessional contexts (Taichman *et al.*, 2012; Kalenderian *et al.*, 2013; D’Assunção *et al.*, 2021). Leaders are in the key role in enabling and supporting effective teamworking, developing and maintaining relationships and creating a culture of trust and openness between all team members through collaborative and shared leadership style (Wali, 2018; Grocock, 2020).

Additionally, it has been found that new graduates need social support and supportive communication to build their professional identities, especially when they are entering working life (Hakkarainen and Mikkola, 2019). Graduating dental students have considered mentorship to be one of the most important factors in job selection after graduating (Stringer and Kerpelman, 2010; Li *et al.*, 2018; Mei *et al.*, 2020). The transition to working life is a major step for any newly graduated dentist (Hakkarainen and Mikkola, 2019), causing stress and uncertainty in both professional and private life (Di Pierro, 2010). Hence, it is important to determine students’ perspectives on leadership and the work community, as knowledge of their views could be used to strengthen the commitment of young professionals and to develop appropriate leadership training. As students will be responsible for shaping the profession in the future, it is beneficial to get their voices heard on the topic. For these reasons, this study aimed to explore what kind of perceptions graduating dental students have on leadership and work communities, and about themselves as leaders and work community members after completing a leadership course tailored for them.

## Materials and methods

The research material consisted of essays written by dental students in their fifth year of education as a final assignment in a leadership course, *Dentist as a Leader*, organized at the University of Eastern Finland during spring 2020. The course is organized annually and is included in the compulsory degree requirements of all students. The goal of this course is to provide soon-to-be graduates with knowledge, attitudes and skills concerning leadership. The course contains lectures, a panel, practical exercises and reflective essay (more detailed description in Table 1). Due to the Covid-19 pandemic, the course had to be modified to fit e-learning platforms on short notice. However, this did not result in any significant changes to the content of the course.

In total, there were 34 participants in this course, forming the target group of the present study. As participation in the study was voluntary, the study group was made up of 30 volunteers (9 male and 21 female participants). Nearly all the participants had gathered work experience during the previous summer, as undergraduate dental students in Finland after completing four years of their education are entitled to temporarily practice as a licensed health-care professional under supervision in oral health services outside their education [Finlex (2023). Health Care Professionals Act 559/1994]. In Finland, the dentist work force is quite evenly divided between private and public sectors. About 18% of the dentists have other dentists as subordinates, Finnish Dental Association (2021). Dentist leaders’ work includes management of everyday functions, personnel and financial management, development tasks and interaction with other health-care units, social services and other sectors, for example, schools. In smaller units, it also includes clinical work. Therefore, we assume that the participants of this study probably got more learning outcomes from the leadership course as they were able to reflect on their previous summer experiences as members of the oral health-care community both during the course and in the essays.

Our study is based on educational theories which highlight the role of reflection in the learning process (Tynjälä *et al.*, 2020; Virtanen and Tynjälä, 2022). Hence, in the essays, the students were asked to include their thoughts about their views and expectations of dentists as leaders and as well as members of the oral health-care work community. They also reflected on how they believed they would meet these expectations. The essay was the final assignment of the course in which students were asked to reflect on their learning experiences of the course freely from their own view. The course assessment was accepted or rejected without numerical grades and was based on participation activity during lessons and practical exercises and the returned essay assignment (Table 1).

The content of the participants’ reflective essays was analyzed using the qualitative content analysis method (Elo and Kyngäs, 2018). The content analysis process enables compressing broad subjects, such as in this study, into a comprehensible format. Before the first phase of our analysis process, the names of the students were removed from the essays to anonymize the data. The names were in the essays only to make sure that students returned the essays. After checking that, the names were removed. So, the data analysis process was completely anonymous.

The first phase of our analysis process included familiarization with the essays written by the participants. The essays were read by three out of the four researchers of this study (MK, MLH, TT). Similar attribute units (words, clauses and sentences) were grouped, and the number of different themes formed was limited by combining similar attributes together and separating distinctive nuances into the same themes (Tuomi and Sarajärvi, 2018). Results are shown according to these themes. Brief quotations from the essays have been attached to the results section to give the reader an opportunity to assess the interpretations made.

During the whole research process, several discussions were held in the research group about the content of the text material and the way the data were categorized based on the themes which students were asked to reflect on. The researchers had continuous, collaborative and multifaceted discussions in which researchers self-consciously evaluated the research project and how their subjectivity and context of the study could influence on the analysis of the data (Olmos-Vega *et al.*, 2022).

Ethical issues were taken into account in this study as follows. All the participants were informed that the use of the essays for research purpose was purely voluntary. In the questionnaire, the students gave their written consent to use the essays for this study. We have followed the Finnish national guidance (Finnish Advisory Board on Research Integrity, 2012) and University of Eastern Finland´s internal instruction of the data processing agreement. In the questionnaire, the participants were informed that their permission was fully optional, and their essays would not be used unless they willingly gave their consent. In the data processing agreement, students got pre-information about the purpose of this study and the data collection, analysis process and disclosure of the data. They had the opportunity to ask questions about the research if they found it necessary. They were informed that their essays will be treated confidentially and will not be disclosed to third parties. They got time to consider their participation in this study.

## Results

### Interpersonal communication competence is an important skill to a leader and a work community member

In their essays, the students wrote their perceptions of leadership and work community mainly from the perspective of social interaction. They evaluated interpersonal communication competence as one of the most important skills to a leader and an oral health-care work community member. As almost all the students had gathered their first work experience as a dentist during the previous summer, they wrote about their views and expectations partly reflecting on these and partly on the course experiences:

A good leader has an ability to change their attitude and interaction according to the personalities of subordinates, which helps to bring out the best sides of the employee and create a harmonious atmosphere and work environment for them. -Student6-

Leadership is working with people, paying attention to people, anticipating things and reacting to them. -Student22-

In the context of leadership, students emphasized emotional and supportive aspects such as listening, empathy and emotional intelligence as well as friendliness and encouragement. The students also brought up skills related to giving positive feedback to employees as well as accepting negative feedback from them. A good leader was most often described as someone who treats employees equally, takes care of the well-being of the workforce and is able to acknowledge employees as individuals. Cooperation, negotiation and problem-solving skills were also described as important qualities for a leader. The students expected a good leader to be assertive and to act responsibly, reliably and purposefully. Openness, flexibility and patience, and the courage to make compromises and decisions, were also mentioned:

On the other hand, (as a leader) you must also be able to accept factual feedback. -Student9-

A good leader is expected to have several different skills, easy approachability, good ethics, good communication and cooperation skills and clear leadership. -Student2-

In addition, some students perceived patient work as a form of leadership although final decision-making power about the treatment was considered to lie at the hands of the patient. They perceived a dentist to have the highest level of knowledge about the treatment options and opportunities, embodying knowledge-based leadership by communicating these to the patient. The importance of smooth cooperation with a dental assistant was also deemed important:

Neither can do their part without the other. In fact, you could describe a good partnership as a kind of symbiosis (a dentist and a dental assistant). -Student23-

Students also reflected on how the leader affects the work community. Opinions were divided: some argued that work community shapes the leader while others considered this to occur in reverse.

[…] a workplace often looks like its leader. -Student5-

I believe that a leader adapts to the work community’s needs. -Student28-

In the context of the work community, what students generally expected from workmates was equal treatment, adherence to rules and an ability to make compromises. Accordingly, a good work atmosphere was frequently mentioned. While the students gave various definitions of a good work atmosphere, many included aspects such as trust in professional skills, respect, giving and receiving help when needed and openness in interaction. Some students mirrored these viewpoints to their first work experiences as dentists, as the following example shows:

They trusted in my skills and […] listened to my wishes. The positive experiences made me feel confident about my competence and motivated me at work. -Student8-

The students also pointed out that the way in which the workplace is organized influences comfort at work. Having explicit norms and rules at the workplace, continuous evaluation and development were said to improve the effectiveness of a work community. Knowing whom to turn to if help was needed, making sure that everyone’s job description is clear to all and ensuring supervisors’ availability were factors that the students expected from a good workplace.

### Students’ self-reflections varied from self-confidence to uncertainty

In relation to the oral health-care work community, most students believed that they would fit in well, mostly as a result of their interpersonal communication competencies. This included getting along with others at work, being able to maintain a positive atmosphere, showing empathy and sticking to mutual rules. Some examples included motivating colleagues by giving positive feedback and cooperating with all the work mates despite possible differences. While some also mentioned that professional roles and expectations could cause anxiety and stress, they represented a small minority:

I feel that particularly young professionals can take on too much pressure due to expectations, which can cause excessive work-related strain. -Student16-

One of the themes surfacing in the students’ essays was their professional identity, which was still taking shape. This was apparent, for instance, as a need for external authority, difficulty in making clinical decisions and being overly flexible (to the point of exhaustion). The students mentioned that they would feel more confident if they were supported by a senior colleague and by sharing their experiences with other students in the same situation:

I still lack confidence in my competence and decision-making. I find myself looking for an authority that I could lean on in decision-making concerning patients. -Student 7-

One key difficulty related to professional identity also concerned assuming the dentist’s leadership role in the dentist–dental assistant interactions:

Some nursing staff members aimed to influence the decisions made on care, usually using the argument “this is the way we have always done this” or “that’s what the other dentist does, why wouldn’t you?” -Student17-

Some students reflected on their previous negative experiences of not getting their views heard, or the atmosphere at their workplace being less than favorable. Most students particularly struggled with voicing their opinions or getting to influence matters at the workplace. Some mentioned difficulties in convincing others to introduce new practices, as more experienced colleagues had been unwilling to change the way they had always worked. Students were aware that a leader is expected to have comprehensive knowledge of economics and the law. However, as the students considered these topics as either unappealing or too far removed from the field of dentistry, they mentioned these as the most prevalent reasons for opting not to seek a leadership position.

### Most of the students expressed changed views on leadership

Most of the students expressed that their views on leadership had changed during the course. Before the module, many had not considered leadership as part of a dentist’s job description or had a very negative, even fearful attitude toward this subject:

For me, just the name of this course, Dentist as a Leader, caused moderate anxiety and despair […]. However, the course ended up increasing my knowledge of what it means to serve as a leader and what makes a good leader. -Student11-

Many of the most negative views of leadership were caused by the students’ perspective of leadership as a personal trait and a fear of not being able to fulfill related expectations.

Ultimately, most of the students had neutral to positive views of leadership and some even mentioned that the course had piqued their interest in the matter. The ones who had already regarded leadership positively at the start of the course mentioned that the course content had further strengthened this view. At the end of the course, positive mindsets related to the development of leadership skills could be perceived, emerging as an interest in pursuing leadership training and obtaining practical knowledge from the field of dentistry:

I have learned to identify my personal strengths as a leader and have also paid attention to areas that I should further develop. -Student31-

Most, if not all, of the students gained knowledge of the diversity of leadership: most mentioned that, at the start of the module, they had perceived leaders as persons in administrative positions and had never perceived those operating in roles such as mentors as also serving as leaders.

Most of the students who were interested in leadership identified their main motivation as having a personal interest in leadership and opportunities for specialization, and an ability to make a more significant impact on issues. Knowledge-based leadership was also mentioned by those who saw themselves specializing further in dentistry. The students also perceived leading projects and setting clear aims as easier than leading people. The difficulties mentioned related to leading people mostly included leading more experienced colleagues with a strong ideal of professional independence as well as leading other dental health professionals (dental hygienists, dental assistants, etc.) whose job content may not be fully clear to the leader:

[…] a young dentist leading older dentists and nursing staff may cause unfounded negative attitudes only because of the young dentist's age, and indignation among the members of the working community. -Student4-

As a young dentist, I also find it challenging to be the leader of an older or more experienced work mate. Do I dare to bring up an opinion that differs from that of a more experienced dentist or dental assistant and make my own decisions despite them? -Student18-

While not all of the participants wanted to pursue higher leadership roles, most participants described they should assume a leadership role in the dentist–dental assistant partnership. Many noted that the dentist carries the ultimate responsibility for the care procedures. Some students mentioned having found it difficult to work with a more experienced assistant who had failed to listen to the student dentist’s wishes and instructions. A strong authoritarian leadership style was not mentioned in this context; instead, the students referred to a kind of symbiotic partnership in which both professionals have their own set of responsibilities and there is no strong role division:

I have experienced the cooperation between a dentist and a dental assistant as teamwork, which works best when there is open discussion and proposals are presented on both sides to optimize working methods. -Student10-

Despite the dentist's leadership position, I would see the dental assistant more as a work mate. Both contribute equally to the patient's treatment with their own work and expertise. -Student12-

## Discussion

In this study, students’ perceptions of leadership were positive after the completion of the course. The course had helped students to realize their own potential in this area. The course provoked students to reflect on their first working life experiences as a dentist, which is a positive result of this study. New graduates need support in building self-confidence and belief in their ability to make an impact in the workplace. Interpersonal communication competence was found to be a major factor in leadership, work community and at the individual level.

Our study showed that the dental students close to graduating valued social factors as the most important aspects of leadership and a good work community. This included issues such as emotional intelligence, empathy, feedback and open interactions and interpersonal communication competence. They emphasized the importance of a good work atmosphere by describing it as trust in professionalism among workmates, mutual respect and giving and receiving help when needed. The results reflect students’ understanding of leadership as collaborative, relational and socially shared task, in which the important goal is to create culture of trust between all members of the work community (Wali, 2018; Grocock, 2020; Hanks, 2020). This means a strong commitment to openness, honesty, inclusiveness and high standards in undertaking the leadership role (Brocklehurst *et al.*, 2013). Students described leadership as a dynamic, socially constructed process, which occurs between work mates demonstrating reciprocal influence on one another, as defined by Hanks *et al.* (2020).

When comparing what our study group appreciated in leadership and work community to previous research findings from different fields and different target groups, the themes found were rather similar (Li *et al.*, 2018; Shu *et al.*, 2018; Ashraf, 2019). This is not surprising since these features are essential to any employees’ job satisfaction as well as their quality of life. However, our research highlighted the students’ need and wish for social support and acceptance, which has been less addressed in previous studies of graduating dentists. Hence, supportive communication could be an appropriate practice to help students to build their professional identities, especially when they are entering working life (Hakkarainen and Mikkola, 2019).

Nearly all students made at least some reference to their professional identity and how this is still in the process of forming. Therefore, we concluded that social support aids career-related decision-making and professional identity commitment, and support provided in the form of a more experienced colleague as a mentor or a close supervisor would certainly help recent graduates, as several studies have expressed (Stringer and Kerpelman, 2010; Hakkarainen and Mikkola, 2019; Keinänen *et al.*, 2020; Mei *et al.*, 2020). A constructive way of action at work is also linked to the way in which newly graduated dentists feel secure to gather experiences, ask for help and grow professionally. Mentorship could also be beneficial for lowering the threshold for students to seek help and consultation. This could promote new graduates’ transition to working life and their quality of life at this stage. Some students mentioned that the high expectations put on new graduates produce stress and feelings of inadequacy. This result is in line with previous studies that have shown that dentists as well as other health-care graduates are experiencing fatigue or stress in the early stages of their careers (Di Pierro, 2010; Hakkarainen and Mikkola, 2019). Therefore, it is essential to improve goal-oriented mentoring practices to sufficiently guide students into the working environment (Keinänen *et al.*, 2020). A good mentor is one of the most important criteria for students when choosing to apply for their first job (Mei *et al.*, 2020).

It is self-evident that we may fail to reach graduates with potential for leadership if they primarily perceive leadership and their own competence negatively. Not every young dentist needs to pursue a high leadership role, but it is important to equip them with knowledge of leadership which is relevant for all work community members. Learning to be assertive and to lead the oral health-care team in a constructive manner are key values of well-functioning cooperation at work (Botelho *et al.*, 2022). At the very least, every dentist will be in charge of an oral health-care team, making decisions on their patient’s treatment and especially leading their own work, ensuring that their knowledge is up-to-date and that they engage in continuous training. This shows the need for leadership courses as without this course, some of the students included in this study may have graduated without accurate knowledge of what is required of them in the area of leadership. Universal leadership course would equip new graduates with knowledge and skills that would enable them to tackle upcoming challenges even better (Kalenderian *et al.*, 2013; Hayes and Ingram, 2019; D’Assunção *et al.*, 2022).

As society is subject to increasingly rapid developmental changes, there is a need for enough competent leaders to rise up to the emerging challenges (Brocklehurst *et al.*, 2013; Widström *et al.*, 2019). This study showed that students’ perceptions of themselves as leaders are often characterized by doubts and, occasionally, even fears. We found that students’ perceptions of leadership could be influenced during the course to become more neutral, perhaps even positive. Without this, some may have ended up with an outdated view of what good leadership is and may not have realized their own potential in this area.

### Value and limitations

The value of this study lies in the fact that graduating dental students’ perceptions concerning leadership and work communities have not been widely explored. The students’ perceptions of leadership were positive after the course and helped them to realize their own potential in this area. The research material obtained from the students’ self-reflecting essays is valid and contains information about the research phenomenon as open questions can generate more in-depth knowledge of students’ perceptions compared to methods such as a survey. However, it is possible that the educational context and the essay writing instructions may have influenced the content of the essays. The study was conducted at the University of Eastern Finland and the findings may not be generalizable across different cultures and educational systems. However, these findings could be utilized in the leadership training of all health-care professionals, but additional research is required.

The essays were read in close detail by three of the four researchers and thus strengthen the reliability of these findings. Making excessive interpretations was avoided, although the study method requires a certain degree of the researchers’ own conceptualization. Several discussions were held about the content of the text material and the way it should be categorized. Brief quotations from the essays were attached to give the reader an opportunity to assess the interpretations made.

## Conclusions and practical implications

Dental students should be equipped with knowledge of leadership and related skills before graduating, as these play a relevant role in any dentist’s work. Dental students need more competence related to leadership that will enable them to, for instance, manage demanding communication situations and influence matters they find important. Students of this study valued social skills as the most important aspects of leadership and good work community such as emotional intelligence, empathy, feedback and open interactions and interpersonal communication competence. They emphasized the importance of a good work atmosphere by describing it as trust in professionalism among workmates, mutual respect and giving and receiving help when needed, social support and acceptance. The course presented in this article did not only have a positive effect on students’ understanding of leadership but also boosted most participants’ self-confidence. In addition, this study produced information on leadership and work communities from students’ perspectives. These findings could be used in developing leadership training as well as in improving daily clinical practice in collaboration between innovative graduates and a supportive work community.

## Figures and Tables

**Table 1.** Course outline and content of each section of the *dentist as a leader* -course

Course outline	Content of the course section
Lectures	• Orientation lecture (background of the course and orientation to later course activities)
20 h	• Lectures/discussions by/with young dentists in specialist education (leading one’s own work and career and working as a dentist)
	• Lectures by dentist leaders from public and private sectors (their careers in leadership, current work, and views of these subjects), and by a leadership professional with background as a dentist (career path, experiences in various leadership roles)
	• Panel discussion with five dentists with specialist education in dental public health (2), currently participating in the training (2) or planning to participate in it (1). The dentists were all at different stages of their leadership careers and had ended up in their positions through different paths
	• Lectures by the Finnish Dental Association and the Finnish Patient Insurance Centre (important cooperation organizations for dentists and dentist leaders)
Preliminary assignments	• Reading articles and literature on topics such as the elements of successful leadership and the development of interpersonal communication competence and reflecting on the themes emerging from the material
Practical exercises	• The first exercise involved reflecting on the students’ own interpersonal communication competence in the role of a leader as well as an employee. The students evaluated their strengths in this area and set goals for improving these skills. The students shared their experiences gathered in work communities with their peers and presented ideas of what kind of leaders they would like to be
6 hours	• In the second exercise, student prepared and gave short speeches to two different target groups (i.e. parents, media). The students recorded their speeches on video and shared them in small groups and evaluated the strengths and areas of development in other students’ speeches
Reflective essay	• The students were asked to write a learning diary during the course about the most relevant and influential topics raised during the lectures and practical exercises and how they could implement these ideas in their own professional lives. Based on this, the students wrote 2–4-page reflective essays on their views of dentists’ roles in the work community and leadership position and how they are able to fill these expectations

**Source:** Authors own work
